# *N*′-(2,6-Di­methyl­phen­yl)-*N*-phenyl­methanimidamide

**DOI:** 10.1107/S2414314625008673

**Published:** 2025-10-28

**Authors:** Ilyes Oubaha, Arindam Saha, Garry S. Hanan, Mihaela Cibian

**Affiliations:** a Département de chimie, Université de Montréal, Complexe des sciences, 1375, Avenue Thérèse-Lavoie-Roux, Montréal, Québec, H2V 0B3, Canada; b Département de biochimie, chimie, physique et science forensique and l’Institut de recherche sur l’hydrogène, Université du Québec à Trois-Rivières, 3351, boul. des Forges, CP 500, Trois-Rivières, Québec, G9A 5H7, Canada; University of Aberdeen, United Kingdom

**Keywords:** crystal structure, acetamidine, hydrogen bonding

## Abstract

The title compound, C_16_H_18_N_2_, is a non-symmetrically *N*,*N*′-disubstituted acetamidine having a phenyl and a bulky 2,6-di­methyl­phenyl as substituents on the two N atoms of the N—C—N linkage. It crystallizes in an *E*-*syn* configuration and its amidine C—N bonds present distinct amine [1.366 (1) Å] and imine [1.288 (1) Å] bond lengths. In the extended structure, strong N—H⋯N hydrogen bonds link the mol­ecules into infinite *C*(4) chains propagating along the *c*-axis direction; weak C—H⋯π inter­actions are also present in the crystal packing.

## Abstract 2

The extended structure of the title compound features strong N—H⋯N hydrogen bonds forming infinite *C*(4) chains propagating along the *c*-axis direction.

## Graphical abstract

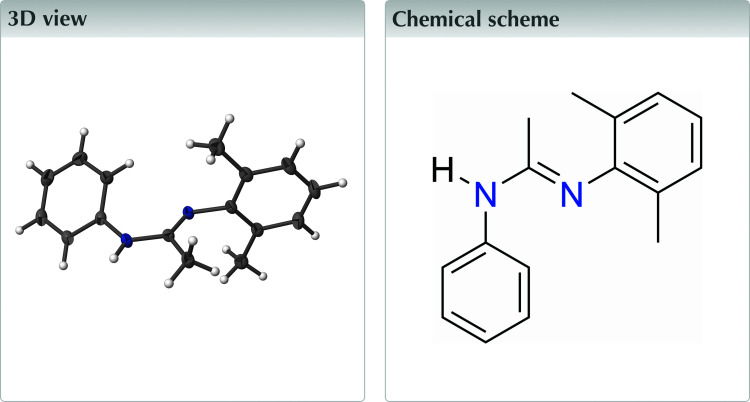

## Structure description

**Figure 1:**
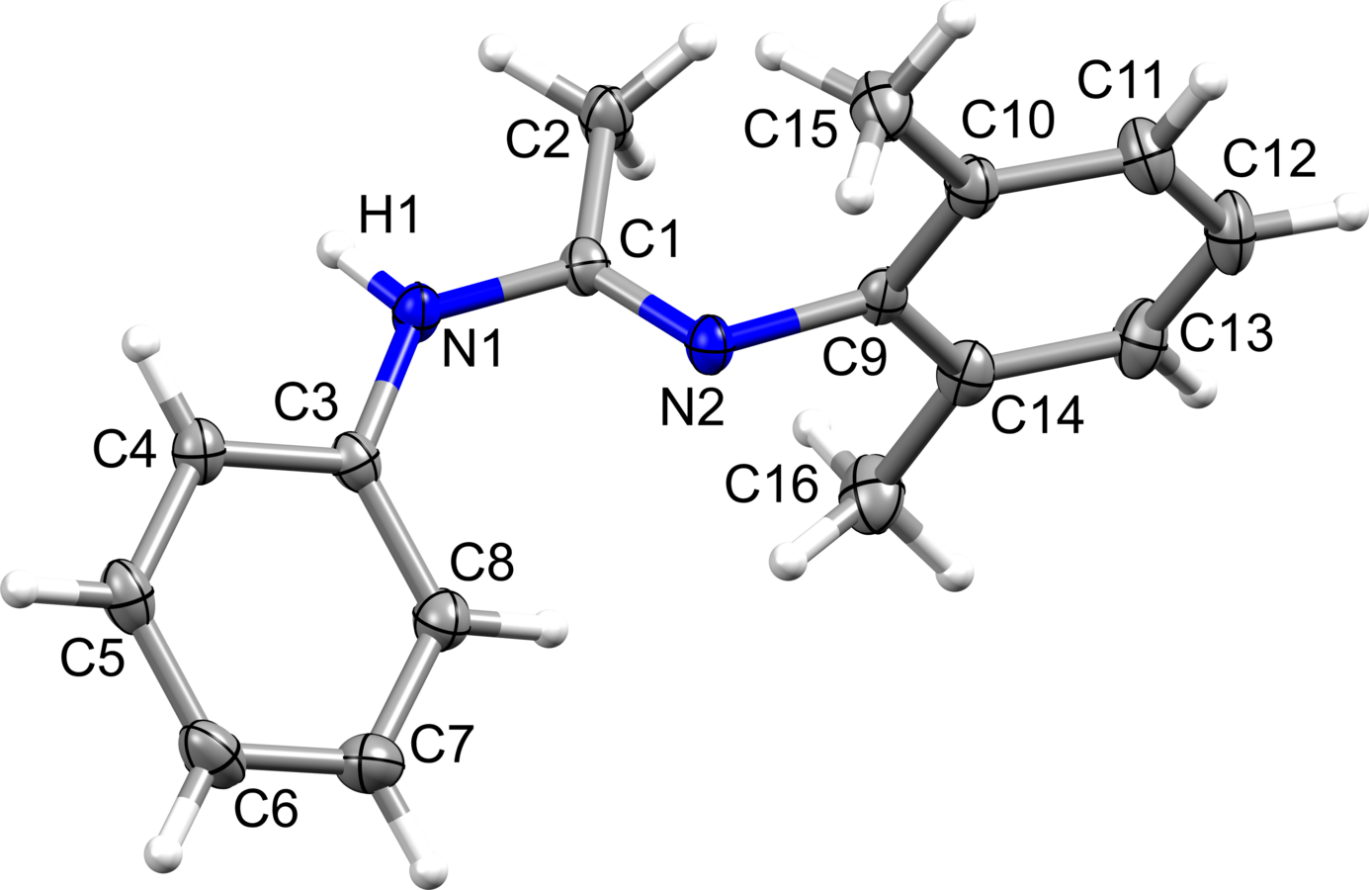
The mol­ecular structure of **1**, with displacement ellipsoids drawn at 50% probability level.

The mol­ecular structure of the title compound, C_16_H_18_N_2_ (**1**) (Fig. 1 ▸) was determined at 100 K. Compound **1** was obtained as a result of a test reaction for synthesizing non-symmetrical amidines under microwave activation by sequential introduction of the *N-*substituents, with the final goal to serve as precursor for the corresponding amidine-*N*-oxide/hy­droxy­amidine derivative (Cibian *et al.*, 2011 ▸; Saha *et al.*, 2024 ▸). Although crystallographic evidence of various non-symmetric acetamidines with *N*,*N*′-bis­aryl­amidines exists (*e.g.*, Stibrany & Potenza, 2007 ▸; Peoples *et al.*, 2012 ▸), this is the first report of **1**, an acetamidine having a phenyl and a bulky 2,6-di­methyl­phenyl as substituents on the two N atoms of the N—C—N linkage. It crystallizes in the monoclinic *P*2_1_/*c* space group, in the *E*-*syn* configuration (Kalz *et al.*, 2016 ▸). Its amidine C—N bonds present distinct amine [1.366 (1) Å] and imine [1.288 (1) Å] bond lengths, as also found for *N*,*N*′-disubstituted aryl­amidine (Boeré *et al.*, 1998 ▸; Cottin *et al.*, 2021 ▸). The phenyl and 2,6-di­methyl­phenyl groups are positioned on the amine and imine N atoms, respectively.

In **1**, the bulky substituted C9–C14 aryl ring and the C3–C8 phenyl ring subtend tilt angles of 85.3 (1) and 40.4 (1)°, respectively, with the N1—C1—N2 plane; the pendant rings are tilted by 64.3 (1)° with respect to each other. The C3—N1—C1—N2 and N1—C1—N2—C9 torsion angles are −11.33 (18) and 176.57 (10)°, respectively.

**Figure 2:**
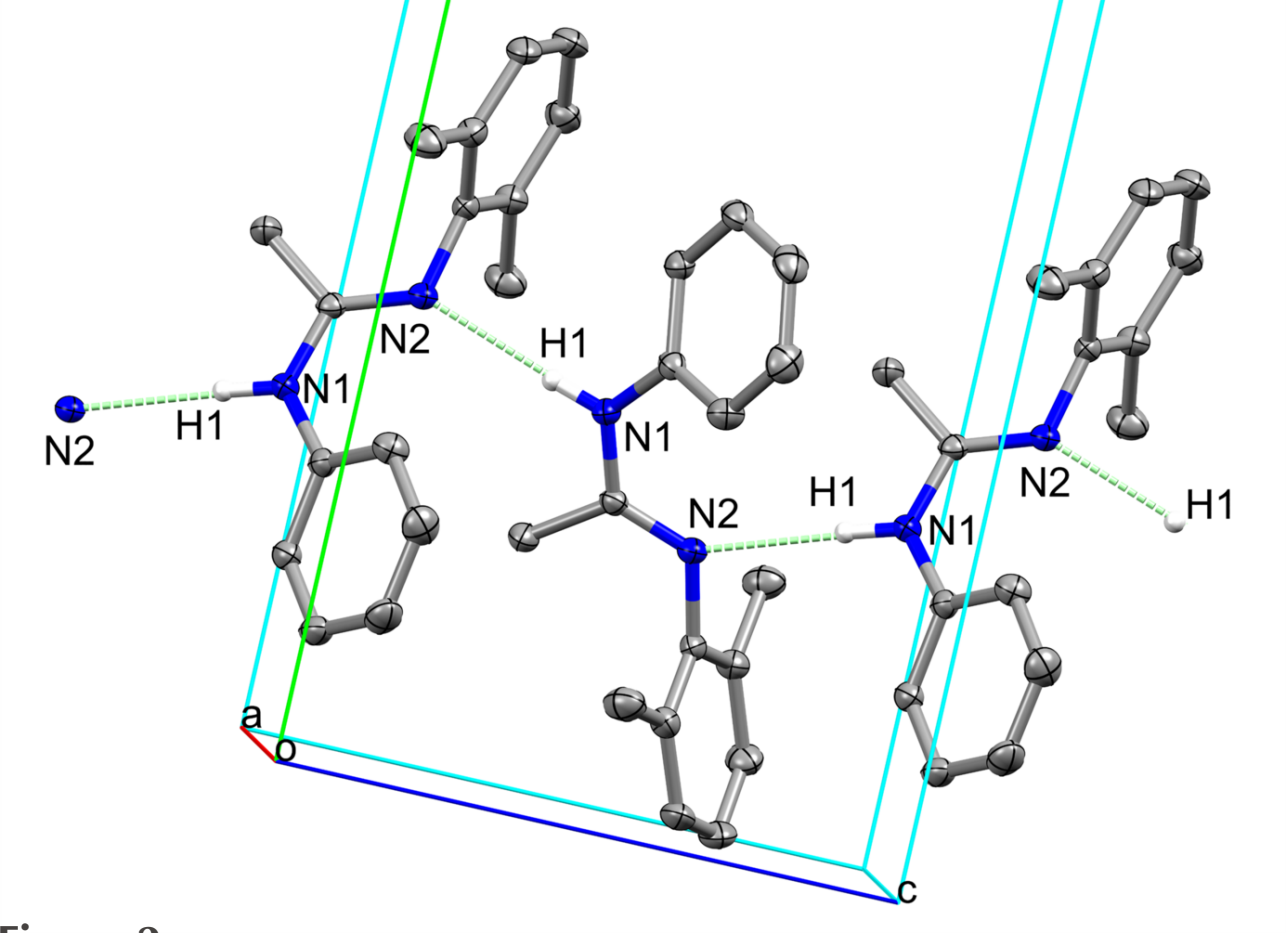
Part of a [001] hydrogen-bonded chain in the extended structure of **1**.

In the extended structure of **1** (Table 1 ▸ and Figs. 2 ▸ and 3 ▸), the mol­ecules are linked by N—H⋯N strong hydrogen bonds (Desiraju & Steiner, 2001 ▸) between the amidine H1 proton and the N2 atom of the amidine unit in an adjacent mol­ecule, thereby forming infinite *C*(4) chains of mol­ecules propagating along the *c-*axis direction. Weak C—H⋯π inter­actions (Desiraju & Steiner, 2001 ▸) complete the crystal packing.

## Synthesis and crystallization

A microwave vial was charged with 600 mg of 4 Å mol­ecular sieves, tri­ethyl­ortho­acetate (1.5 ml, 8.18 mmol, 1 equiv.), 2 drops of acetic acid, and aniline (0.75 ml, 8.18 mmol, 1 equiv.). The reaction was conducted under microwave irradiation at 90 °C for 10 min. After cooling down, 2,6-di­methyl­anilline (1.01 ml, 8.18 mmol, 1 equiv.) was added to the crude reaction mixture and the reaction was continued at 90 °C for another 16 h. Part of the reaction mixture was taken in hexane (the mol­ecular sieves were removed by filtration) and the solution was placed in the freezer (−10 °C). XRD-quality colorless crystals were obtained (0.50 g, 2.10 mmol, partial yield: 26% – only part of the product was purified).

^1^H NMR (400 MHz, DMSO-*d_6_*): 1.69 (*s*, 3H); 1.99 (*s*, 6H), 6.76 (*t*, *J* = 7.5 Hz, 1H), 6.91 (*t*, *J* = 7.3 Hz, 1H), 6.98 (*d*, *J* = 7.5 Hz, 2H), 7.25 (*t*, *J* = 7.5 Hz, 2H), 7.82 (*d, J* =7.9 Hz, 2H), 8.87 (*s*, 1H, NH). ^13^C NMR (100 MHz, DMSO-*d_6_*): 17.9, 18.0, 118.7, 121.0, 121.1, 127.0, 127.5, 128.2, 141.4, 148.5, 152.2 (N—C=N). Elemental analysis C/H/N: calculated (%) for C_16_H_18_N_2_: C 80.63, H 7.61, N 11.75; exp .: C 80.57, H 7.56, N 11.81. HRMS (ESI, positive): *m*/*z* [*M* + H]^+^ calculated: 239.15428; exp .: 239.15398 (diff. 1.25 p.p.m.).

## Refinement

Crystal data, data collection and structure refinement details are summarized in Table 2 ▸.

## Supplementary Material

Crystal structure: contains datablock(s) I. DOI: 10.1107/S2414314625008673/hb4536sup1.cif

Structure factors: contains datablock(s) I. DOI: 10.1107/S2414314625008673/hb4536Isup2.hkl

Supporting information file. DOI: 10.1107/S2414314625008673/hb4536Isup3.cml

CCDC reference: 2492957

Additional supporting information:  crystallographic information; 3D view; checkCIF report

## Figures and Tables

**Figure 3:**
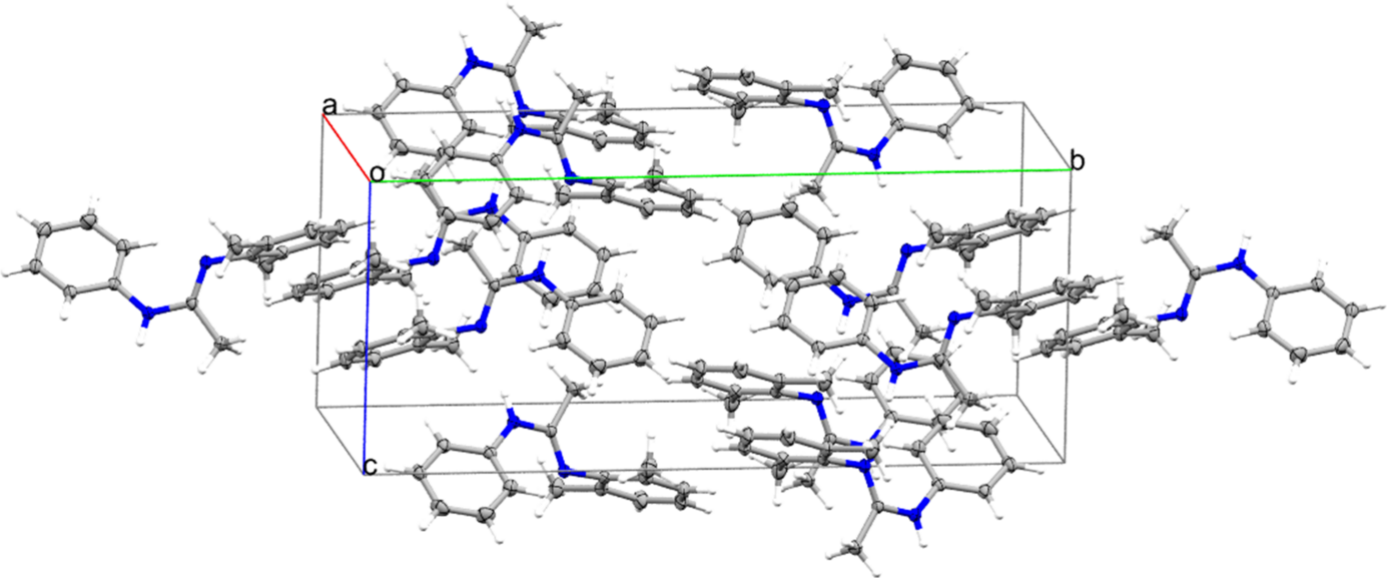
View of the crystal packing of **1** in the unit cell.

**Table 1:** Hydrogen-bond geometry (Å, °) *Cg*1 and *Cg*2 are the centroids of the C3–C8 phenyl and C9–C14 2,6-di­methyl­phenyl rings, respectively.

*D*—H⋯*A*	*D*—H	H⋯*A*	*D*⋯*A*	*D*—H⋯*A*
N1—H1⋯N2^i^	0.882 (15)	2.172 (15)	3.0530 (14)	176.3 (14)
C2—H2*C*⋯*Cg*1^i^	0.98	2.83	3.5601 (14)	132
C4—H4⋯*Cg*2^i^	0.95	2.59	3.5092 (13)	162
C15—H15*A*⋯*Cg*1^ii^	0.98	2.83	3.6346 (15)	140

Symmetry codes: (i) 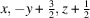; (ii) 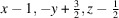.

**Table 2:** Experimental details

Crystal data	
Chemical formula	C_16_H_18_N_2_
*M*_r_	238.32
Crystal system, space group	Monoclinic, *P*2_1_/*c*
Temperature (K)	100
*a*, *b*, *c* (Å)	7.2094 (1), 21.3517 (4), 8.9033 (2)
β (°)	95.418 (1)
*V* (Å^3^)	1364.39 (4)
*Z*	4
Radiation type	Cu *K*α
μ (mm^−1^)	0.53
Crystal size (mm)	0.21 × 0.04 × 0.01 × 0.01 (radius)

Data collection	
Diffractometer	Bruker SMART APEXII CCD
Absorption correction	For a sphere (*SADABS*; Krause *et al.*, 2015 ▸)
*T*_min_, *T*_max_	0.807, 0.993
No. of measured, independent and observed [*I* > 2σ(*I*)] reflections	21279, 2595, 2160
*R*_int_	0.030
(sin θ/λ)_max_ (Å^−1^)	0.613

Refinement	
*R*[*F*^2^ > 2σ(*F*^2^)], *wR*(*F*^2^), *S*	0.038, 0.113, 1.08
No. of reflections	2595
No. of parameters	170
H-atom treatment	H atoms treated by a mixture of independent and constrained refinement
Δρ_max_, Δρ_min_ (e Å^−3^)	0.28, −0.27

Computer programs: *APEX2* (Bruker, 2009 ▸), *SAINT* (Bruker, 2020 ▸), *SHELXT* (Sheldrick, 2015*a* ▸), *SHELXL* (Sheldrick, 2015*b* ▸), *OLEX2* (Dolomanov *et al.*, 2009 ▸), *ORTEP-3 for Windows* (Farrugia, 2012 ▸), *publCIF* (Westrip, 2010 ▸), *POV-RAY* (POVRAY, 2013 ▸), *PLATON* (Spek, 2020 ▸) and *Mercury* (Macrae *et al.*, 2020 ▸).

## full crystallographic data

### N'-(2,6-Dimethylphenyl)-N-phenylmethanimidamide Crystal data

**Table:**

C_16_H_18_N_2_	*F*(000) = 512
*M**_r_* = 238.32	*D*_x_ = 1.160 Mg m^−^^3^
Monoclinic, *P*2_1_/*c*	Cu *K*α radiation, λ = 1.54178 Å
*a* = 7.2094 (1) Å	Cell parameters from 7241 reflections
*b* = 21.3517 (4) Å	θ = 4.1–70.8°
*c* = 8.9033 (2) Å	µ = 0.53 mm^−^^1^
β = 95.418 (1)°	*T* = 100 K
*V* = 1364.39 (4) Å^3^	Needle, colourless
*Z* = 4	0.21 × 0.04 × 0.01 × 0.01 (radius) mm

### N'-(2,6-Dimethylphenyl)-N-phenylmethanimidamide Data collection

**Table:**

Bruker SMART APEXII CCD diffractometer	2595 independent reflections
Radiation source: microfocus sealed X-ray tube, Incoatec Iµs	2160 reflections with *I* > 2σ(*I*)
Mirror optics monochromator	*R*_int_ = 0.030
Detector resolution: 7.9 pixels mm^-1^	θ_max_ = 71.0°, θ_min_ = 4.1°
ω and φ scans	*h* = −8→8
Absorption correction: for a sphere (SADABS; Krause *et al.*, 2015)	*k* = −26→26
*T*_min_ = 0.807, *T*_max_ = 0.993	*l* = −10→10
21279 measured reflections	

### N'-(2,6-Dimethylphenyl)-N-phenylmethanimidamide Refinement

**Table:**

Refinement on *F*^2^	Primary atom site location: dual
Least-squares matrix: full	Hydrogen site location: mixed
*R*[*F*^2^ > 2σ(*F*^2^)] = 0.038	H atoms treated by a mixture of independent and constrained refinement
*wR*(*F*^2^) = 0.113	*w* = 1/[σ^2^(*F*_o_^2^) + (0.0716*P*)^2^ + 0.0724*P*] where *P* = (*F*_o_^2^ + 2*F*_c_^2^)/3
*S* = 1.08	(Δ/σ)_max_ < 0.001
2595 reflections	Δρ_max_ = 0.28 e Å^−^^3^
170 parameters	Δρ_min_ = −0.27 e Å^−^^3^
0 restraints	

### N'-(2,6-Dimethylphenyl)-N-phenylmethanimidamide Special details

**Table:**

Geometry. All esds (except the esd in the dihedral angle between two l.s. planes) are estimated using the full covariance matrix. The cell esds are taken into account individually in the estimation of esds in distances, angles and torsion angles; correlations between esds in cell parameters are only used when they are defined by crystal symmetry. An approximate (isotropic) treatment of cell esds is used for estimating esds involving l.s. planes.
Refinement. H atoms were included in calculated positions (C—H = 0.95–0.98 Å) and treated as riding atoms with *U*_iso_(H) = 1.2*U*_eq_(C) or 1.5*U*_eq_(methyl C). The NH proton (H1) was located in the difference-Fourier map and refined freely.

### N'-(2,6-Dimethylphenyl)-N-phenylmethanimidamide Fractional atomic coordinates and isotropic or equivalent isotropic displacement parameters (Å^2^)

**Table:**

	*x*	*y*	*z*	*U*_iso_*/*U*_eq_	
N1	0.67938 (14)	0.76538 (4)	1.09532 (11)	0.0191 (2)	
H1	0.660 (2)	0.7773 (7)	1.1875 (16)	0.030 (4)*	
N2	0.62606 (14)	0.68830 (4)	0.91279 (11)	0.0180 (2)	
C1	0.61447 (16)	0.70782 (5)	1.04845 (12)	0.0174 (3)	
C2	0.52870 (19)	0.67095 (6)	1.16869 (13)	0.0233 (3)	
H2A	0.417882	0.648897	1.123623	0.035*	
H2B	0.493168	0.699538	1.247175	0.035*	
H2C	0.619298	0.640440	1.213416	0.035*	
C3	0.79195 (17)	0.80654 (5)	1.01846 (12)	0.0185 (3)	
C4	0.77137 (17)	0.87086 (5)	1.04025 (13)	0.0209 (3)	
H4	0.677614	0.885791	1.099341	0.025*	
C5	0.88698 (18)	0.91300 (6)	0.97613 (14)	0.0252 (3)	
H5	0.873702	0.956622	0.993177	0.030*	
C6	1.02225 (19)	0.89181 (6)	0.88702 (14)	0.0295 (3)	
H6	1.101809	0.920649	0.843217	0.035*	
C7	1.03948 (19)	0.82817 (7)	0.86297 (15)	0.0298 (3)	
H7	1.130352	0.813541	0.800713	0.036*	
C8	0.92670 (17)	0.78530 (6)	0.92795 (14)	0.0237 (3)	
H8	0.941096	0.741724	0.910947	0.028*	
C9	0.56482 (17)	0.62633 (5)	0.87387 (12)	0.0184 (3)	
C10	0.38863 (17)	0.61776 (6)	0.79602 (13)	0.0218 (3)	
C11	0.33856 (19)	0.55810 (6)	0.74243 (14)	0.0274 (3)	
H11	0.219225	0.551720	0.689757	0.033*	
C12	0.4601 (2)	0.50815 (6)	0.76486 (15)	0.0299 (3)	
H12	0.424966	0.467915	0.726479	0.036*	
C13	0.6326 (2)	0.51699 (6)	0.84329 (14)	0.0267 (3)	
H13	0.714719	0.482358	0.859959	0.032*	
C14	0.68861 (17)	0.57583 (6)	0.89848 (13)	0.0220 (3)	
C15	0.25781 (19)	0.67238 (6)	0.76836 (16)	0.0292 (3)	
H15A	0.134837	0.657116	0.728148	0.044*	
H15B	0.306460	0.700987	0.695456	0.044*	
H15C	0.246968	0.694588	0.863457	0.044*	
C16	0.87995 (19)	0.58474 (6)	0.97859 (16)	0.0314 (3)	
H16A	0.963149	0.551898	0.947325	0.047*	
H16B	0.873019	0.582271	1.087825	0.047*	
H16C	0.928376	0.625855	0.952801	0.047*	

### N'-(2,6-Dimethylphenyl)-N-phenylmethanimidamide Atomic displacement parameters (Å^2^)

**Table:**

	*U*^11^	*U*^22^	*U*^33^	*U*^12^	*U*^13^	*U*^23^
N1	0.0265 (5)	0.0145 (5)	0.0167 (5)	−0.0016 (4)	0.0034 (4)	−0.0011 (4)
N2	0.0220 (5)	0.0134 (5)	0.0184 (5)	−0.0002 (4)	0.0011 (4)	0.0001 (4)
C1	0.0194 (6)	0.0135 (6)	0.0189 (6)	0.0017 (4)	0.0004 (4)	0.0014 (4)
C2	0.0329 (7)	0.0174 (6)	0.0201 (6)	−0.0040 (5)	0.0052 (5)	−0.0003 (4)
C3	0.0209 (6)	0.0173 (6)	0.0166 (6)	−0.0025 (4)	−0.0019 (5)	0.0011 (4)
C4	0.0260 (6)	0.0174 (6)	0.0187 (6)	−0.0006 (5)	−0.0007 (5)	−0.0001 (4)
C5	0.0333 (7)	0.0177 (6)	0.0236 (6)	−0.0055 (5)	−0.0027 (5)	0.0023 (4)
C6	0.0324 (7)	0.0292 (7)	0.0269 (7)	−0.0119 (6)	0.0027 (6)	0.0040 (5)
C7	0.0263 (7)	0.0335 (8)	0.0307 (7)	−0.0045 (6)	0.0081 (6)	−0.0017 (5)
C8	0.0237 (6)	0.0200 (6)	0.0275 (6)	0.0004 (5)	0.0027 (5)	−0.0014 (5)
C9	0.0261 (6)	0.0148 (6)	0.0148 (6)	−0.0012 (5)	0.0044 (5)	0.0005 (4)
C10	0.0262 (6)	0.0171 (6)	0.0223 (6)	−0.0005 (5)	0.0026 (5)	−0.0002 (4)
C11	0.0314 (7)	0.0216 (7)	0.0278 (7)	−0.0049 (5)	−0.0041 (5)	−0.0020 (5)
C12	0.0444 (8)	0.0156 (6)	0.0287 (7)	−0.0032 (6)	−0.0006 (6)	−0.0034 (5)
C13	0.0396 (8)	0.0150 (6)	0.0251 (6)	0.0051 (5)	0.0007 (6)	0.0007 (5)
C14	0.0292 (7)	0.0184 (6)	0.0184 (6)	0.0016 (5)	0.0022 (5)	0.0019 (4)
C15	0.0257 (7)	0.0249 (7)	0.0361 (8)	0.0019 (5)	−0.0023 (6)	−0.0032 (5)
C16	0.0322 (7)	0.0240 (7)	0.0368 (8)	0.0059 (5)	−0.0036 (6)	−0.0018 (5)

### N'-(2,6-Dimethylphenyl)-N-phenylmethanimidamide Geometric parameters (Å, º)

**Table:**

N1—H1	0.882 (15)	C8—H8	0.9500
N1—C1	1.3663 (15)	C9—C10	1.4004 (17)
N1—C3	1.4157 (15)	C9—C14	1.4036 (16)
N2—C1	1.2879 (14)	C10—C11	1.3957 (17)
N2—C9	1.4272 (14)	C10—C15	1.5055 (17)
C1—C2	1.5078 (15)	C11—H11	0.9500
C2—H2A	0.9800	C11—C12	1.3826 (19)
C2—H2B	0.9800	C12—H12	0.9500
C2—H2C	0.9800	C12—C13	1.3810 (19)
C3—C4	1.3967 (16)	C13—H13	0.9500
C3—C8	1.3952 (17)	C13—C14	1.3946 (17)
C4—H4	0.9500	C14—C16	1.5040 (18)
C4—C5	1.3857 (17)	C15—H15A	0.9800
C5—H5	0.9500	C15—H15B	0.9800
C5—C6	1.3897 (19)	C15—H15C	0.9800
C6—H6	0.9500	C16—H16A	0.9800
C6—C7	1.3830 (19)	C16—H16B	0.9800
C7—H7	0.9500	C16—H16C	0.9800
C7—C8	1.3865 (18)		
			
C1—N1—H1	117.7 (10)	C10—C9—N2	119.12 (10)
C1—N1—C3	127.32 (10)	C10—C9—C14	120.70 (11)
C3—N1—H1	114.6 (10)	C14—C9—N2	119.79 (11)
C1—N2—C9	118.81 (9)	C9—C10—C15	120.32 (11)
N1—C1—C2	113.86 (10)	C11—C10—C9	118.79 (11)
N2—C1—N1	121.80 (10)	C11—C10—C15	120.88 (12)
N2—C1—C2	124.34 (10)	C10—C11—H11	119.5
C1—C2—H2A	109.5	C12—C11—C10	120.98 (12)
C1—C2—H2B	109.5	C12—C11—H11	119.5
C1—C2—H2C	109.5	C11—C12—H12	120.1
H2A—C2—H2B	109.5	C13—C12—C11	119.72 (12)
H2A—C2—H2C	109.5	C13—C12—H12	120.1
H2B—C2—H2C	109.5	C12—C13—H13	119.4
C4—C3—N1	118.03 (11)	C12—C13—C14	121.21 (12)
C8—C3—N1	122.65 (11)	C14—C13—H13	119.4
C8—C3—C4	119.25 (11)	C9—C14—C16	121.20 (11)
C3—C4—H4	119.8	C13—C14—C9	118.58 (12)
C5—C4—C3	120.35 (12)	C13—C14—C16	120.20 (11)
C5—C4—H4	119.8	C10—C15—H15A	109.5
C4—C5—H5	119.8	C10—C15—H15B	109.5
C4—C5—C6	120.37 (12)	C10—C15—H15C	109.5
C6—C5—H5	119.8	H15A—C15—H15B	109.5
C5—C6—H6	120.4	H15A—C15—H15C	109.5
C7—C6—C5	119.10 (12)	H15B—C15—H15C	109.5
C7—C6—H6	120.4	C14—C16—H16A	109.5
C6—C7—H7	119.4	C14—C16—H16B	109.5
C6—C7—C8	121.28 (12)	C14—C16—H16C	109.5
C8—C7—H7	119.4	H16A—C16—H16B	109.5
C3—C8—H8	120.2	H16A—C16—H16C	109.5
C7—C8—C3	119.62 (12)	H16B—C16—H16C	109.5
C7—C8—H8	120.2		
			
N1—C3—C4—C5	175.50 (11)	C5—C6—C7—C8	−1.1 (2)
N1—C3—C8—C7	−176.26 (11)	C6—C7—C8—C3	0.5 (2)
N2—C9—C10—C11	172.39 (10)	C8—C3—C4—C5	−1.79 (17)
N2—C9—C10—C15	−6.41 (17)	C9—N2—C1—N1	176.57 (10)
N2—C9—C14—C13	−172.50 (10)	C9—N2—C1—C2	−4.01 (17)
N2—C9—C14—C16	5.76 (17)	C9—C10—C11—C12	−0.21 (19)
C1—N1—C3—C4	149.38 (11)	C10—C9—C14—C13	0.23 (18)
C1—N1—C3—C8	−33.43 (18)	C10—C9—C14—C16	178.49 (11)
C1—N2—C9—C10	100.22 (13)	C10—C11—C12—C13	1.0 (2)
C1—N2—C9—C14	−86.95 (14)	C11—C12—C13—C14	−1.1 (2)
C3—N1—C1—N2	−11.33 (18)	C12—C13—C14—C9	0.53 (19)
C3—N1—C1—C2	169.20 (11)	C12—C13—C14—C16	−177.75 (12)
C3—C4—C5—C6	1.26 (18)	C14—C9—C10—C11	−0.38 (18)
C4—C3—C8—C7	0.89 (18)	C14—C9—C10—C15	−179.18 (11)
C4—C5—C6—C7	0.17 (19)	C15—C10—C11—C12	178.58 (12)

### N'-(2,6-Dimethylphenyl)-N-phenylmethanimidamide Hydrogen-bond geometry (Å, º)

*Cg*1 and *Cg*2 are the centroids of the C3–C8 phenyl and C9–C14 2,6-dimethylphenyl rings, respectively.

**Table:**

*D*—H···*A*	*D*—H	H···*A*	*D*···*A*	*D*—H···*A*
N1—H1···N2^i^	0.882 (15)	2.172 (15)	3.0530 (14)	176.3 (14)
C2—H2*C*···*Cg*1^i^	0.98	2.83	3.5601 (14)	132
C4—H4···*Cg*2^i^	0.95	2.59	3.5092 (13)	162
C15—H15*A*···*Cg*1^ii^	0.98	2.83	3.6346 (15)	140

Symmetry codes: (i) *x*, −*y*+3/2, *z*+1/2; (ii) *x*−1, −*y*+3/2, *z*−1/2.
